# Targeted Sequencing of Alzheimer Disease Genes in African Americans Implicates Novel Risk Variants

**DOI:** 10.3389/fnins.2018.00592

**Published:** 2018-08-27

**Authors:** Mark W. Logue, Daniel Lancour, John Farrell, Irina Simkina, M. Daniele Fallin, Kathryn L. Lunetta, Lindsay A. Farrer

**Affiliations:** ^1^National Center for Posttraumatic Stress Disorder (PTSD), United States Department of Veterans Affairs, Boston Healthcare System, Boston, MA, United States; ^2^Department of Psychiatry, Boston University School of Medicine, Boston University, Boston, MA, United States; ^3^Biomedical Genetics, Department of Medicine, Boston University School of Medicine, Boston University, Boston, MA, United States; ^4^Department of Biostatistics, Boston University School of Public Health, Boston University, Boston, MA, United States; ^5^Department of Mental Health, Johns Hopkins Bloomberg School of Public Health, Johns Hopkins University, Baltimore, MD, United States; ^6^Departments of Neurology and Ophthalmology, Boston University School of Medicine, Boston University, Boston, MA, United States; ^7^Department of Epidemiology, Boston University School of Public Health, Boston, MA, United States

**Keywords:** Alzheimer disease, sequencing, *AKAP9*, *ABCA7*, rare variant, African Americans

## Abstract

The genetic architecture of late-onset Alzheimer disease (AD) in African Americans (AAs) differs from that in persons of European ancestry. In addition to *APOE*, genome-wide association studies (GWASs) of AD in AA samples have implicated *ABCA7*, *COBL*, and *SLC10A2* as AA-AD risk genes. Previously, we identified by whole exome sequencing a small number of AA AD cases and subsequent genotyping in a large AA sample of AD cases and controls association of AD risk with a pair of rare missense variants in *AKAP9*. In this study, we performed targeted deep sequencing (including both introns and exons) of approximately 100 genes previously linked to AD or AD-related traits in an AA cohort of 489 AD cases and 472 controls to find novel AD risk variants. We observed association with an 11 base-pair frame-shift loss-of-function (LOF) variant in *ABCA7* (rs567222111) for which the evidence was bolstered when combined with data from a replication AA cohort of 484 cases and 484 controls (*OR* = 2.42, *p* = 0.022). We also found association of AD with a rare 9 bp deletion (rs371245265) located very close to the *AKAP9* transcription start site (rs371245265, *OR* = 10.75, *p* = 0.0053). The most significant findings were obtained with a rare protective variant in *F5* (*OR* = 0.053, *p* = 6.40 × 10^-5^), a gene that was previously associated with a brain MRI measure of hippocampal atrophy, and two common variants in *KIAA0196* (*OR* = 1.51, *p*<8.6 × 10^-5^). Gene-based tests of aggregated rare variants yielded several nominally significant associations with *KANSL1*, *CNN2*, and *TRIM35*. Although no associations passed multiple test correction, our study adds to a body of literature demonstrating the utility of examining sequence data from multiple ethnic populations for discovery of new and impactful risk variants. Larger sample sizes will be needed to generate well-powered epidemiological investigations of rare variation, and functional studies are essential for establishing the pathogenicity of variants identified by sequencing.

## Introduction

Studies of common genetic variants have identified many gene loci that influence risk of late-onset Alzheimer disease (AD) in persons of European ancestry (EA), most notably the *APOE* 𝜀2 and 𝜀4 alleles which confer strong protective and deleterious effects, respectively (Saunders et al., 1993; Corder et al., 1994), as well as more than 20 modest effect loci (odds ratios between 1.1 and 1.3) including *BIN1*, *CR1*, *ABCA7*, *CLU*, *PICALM*, and the *MS4A* gene region (Lambert et al., 2013). Extensions of these findings and the contributions of additional loci have emerged from investigations of non-EA cohorts, African Americans (AAs) in particular (Reitz et al., 2013a; Mez et al., 2017). The risk of AD is greater in AAs than EAs, however, paradoxically, 𝜀4 has a weaker effect in AAs than EAs (Farrer et al., 1997; Reitz et al., 2013a). These observations and greater genetic diversity among persons with African ancestry suggest that the genetic architecture for AD includes some variants and loci that differ from EAs. Several genome-wide association studies (GWAS) in AAs (Logue et al., 2011; Kamboh et al., 2012; Reitz et al., 2013a) confirmed the role of several genes identified initially in EAs, most notably *APOE* and *ABCA7*. The association peak in *ABCA7* is ascribed to different SNPs in EAs (rs4147929) and AAs (rs115550680; Lambert et al., 2013; Reitz et al., 2013a). Gene resequencing studies have revealed multiple rare *ABCA7* deletions causing missense loss-of-function (LOF) mutations in EAs (Cukier et al., 2016; N’Songo et al., 2017). Cukier et al. (2016) identified a 44 base pair (bp) frameshift deletion in *ABCA7* (rs142076058) that is in linkage disequilibrium (LD) with rs115550680 and thus may be the functional variant underlying the observed association. A recent exome sequencing investigation in an AA cohort of 198 AD cases and 304 controls examined 20 putative AD risk genes implicated by GWAS in EAs, and found nominally (uncorrected) significant associations with two *ABCA7* variants (rs3764647 and rs3752239) and with gene-based tests of coding variants in *MS4A6A*, *PTK2B*, and *ZCWPW1* (N’Songo et al., 2017).

Novel AD loci have been identified in other studies of AA samples. Mez et al. (2017) identified GWAS significant associations with SNPs in *COBL (rs112404845)* and *SLC10A2 (rs16961023)* in a GWAS using an informed conditioning approach. A WES study of seven AA cases followed by genotyping using a staged design in AA cohorts containing 422 cases and 394 controls (stage 1) and 1,037 cases and 1,869 controls (stage 2) identified association with two rare AA-specific highly correlated variants in *AKAP9*, rs144662445 (*OR* = 2.75) and rs149979685 (*OR* = 3.61) (Logue et al., 2014).

These studies confirm the utility of examining African-descent samples to identify new AD risk variants in known AD genes as well as novel AD loci. In this study, we performed targeted sequencing in a discovery cohort containing approximately 1,000 AAs to identify new potentially causal variants in risk genes previously implicated in AD risk in AAs (*ABCA7*, *AKAP9*, *COBL*, *MS4A6A*, *PTK2B*, *SLC10A2*, and *ZCWPW1*) or in AD and related traits in other populations.

## Materials and Methods

### Samples

The targeted gene sequencing sample included AA subjects primarily from two cohorts: the Multi-Institutional Research on Alzheimer Genetic Epidemiology (MIRAGE, 113 AD cases, 131 controls) Study (Green et al., 2002) and the Genetic and Environmental Risk Factors for Alzheimer Disease Among African Americans (GenerAAtions, 222 AD cases, 190 controls) Study (Logue et al., 2011). MIRAGE is a family-based study of clinic-based AD cases and their first-degree relatives. The GenerAAtions study includes unrelated individuals ascertained through the Henry Ford Health System. In addition, we obtained DNA samples and phenotypic data from the National Cell Repository for Alzheimer Disease (NCRAD) that were aggregated from the Ibadan/Indianapolis (INDY) study (Hendrie et al., 1995; Sahota et al., 1997; Gureje et al., 2006), the African American Alzheimer’s Disease Genetics (AAG) study (Meier et al., 2012), the National Institute on Aging Alzheimer’s Disease Centers (ADC) (Jun et al., 2010), and the National Institute on Aging Late-Onset Alzheimer’s Disease (NIA-LOAD) Family Study (Lee et al., 2008). The Indianapolis/Ibadan study comprises elderly AA residents from Indianapolis (community dwelling and nursing home residents) and African-descent residents of Ibadan, Nigeria. The AAG study and ADC cohort include cases and controls ascertained at more than 30 sites across the United States. The NIA-LOAD Study includes families with multiple AD cases and unaffected members and an independent set of cognitively screened controls.

The discovery cohort included 489 cases and 472 controls from the MIRAGE and GenerAAtions studies supplemented with 154 cases and 151 controls from the AAG and Ibadan studies. The replication cohort consisted of additional samples from the AAG, ADC, Indy/Ibadan, and NIA-LOAD studies (484 AD cases, 484 controls). Characteristics of the discovery and replication cohorts are presented in **Table 1**. Further details about subject ascertainment and classification, including genetic screening for ancestry mismatches, were reported elsewhere (Reitz et al., 2013a). The diagnosis of AD in all cohorts was made according to established criteria (McKhann et al., 2011) and all controls were screened to be cognitively normal.

**Table 1:** Sample size and demographics for discovery and replication cohorts.

Discovery Data	Site	Sample size	Cases			Controls		
			*N* (%)	*N* male (%)	mean age at onset (*SD*)	*N* (%)	*N* male (%)	mean age at exam (*SD*)
	MIRAGE	244	113 (46.31%)	27 (23.89)	71.14 (9.25)	131 (53.69%)	40 (30.53)	69.77 (10.16)
	GenerAAtions	412	222 (46.12%)	97 (43.69)	77.27 (6.64)	190 (53.88%)	77 (40.53)	78.38 (6.51)
	Ibadan	119	60 (50.42)	8 (13.33)	84.62 (7.37)	59 (49.58)	29 (49.15)	94.4^∗^ (3.2)
	AAG	186	94 (50.54)	26 (27.66)	80.44 (5.04)	92 (49.46)	14 (15.22)	79.89 (3.34)
	Total discovery	961	489 (50.83)			472 (49.17)		

**Replication Data**	**Cohort**	**Sample size**	**Cases**			**Controls**		
			***N* (%)**	***n* male (%)**	**mean age at onset (*SD*)**	***N* (%)**	***N* male (%)**	**mean age at exam (*SD*)**

	AAG	183	49 (26.7)	13 (26.53)	68.08 (3.81)	134 (73.22)	28 (20.9)	72.52 (2.35)
	ADC	89	73 (82.02)	26 (35.62)	76.22 (6.92)	16 (17.98)	5 (31.25)	77.31 (7.46)
	Ibadan	38	19 (50.00)	3 (15.79)	78.48 (6.30)	19 (50.00)	6 (31.58)	91.45^∗^ (4.23)
	INDY	354	171 (48.31)	58 (33.92)	84.44 (6.53)	183 (51.69)	38 (20.77)	93.81^∗^ (2.9)
	NIALOAD	304	172 (56.58)	61 (35.47)	77.11 (7.83)	132 (43.42)	45 (34.09)	72.6 (7.82)
	Total replication	968	484 (50.00)			484 (50.00)		


^∗^*Controls selected to have a high mean age.*

### Sequencing Methods

The samples in the discovery cohort were sent to the McDonnell Genome Institute at Washington University^1^ for targeted sequencing. The targeted regions included genes previously associated with AD in AAs (*ABCA7*, *AKAP9*, *COBL*, *MS4A6A*, *PTK2B*, *SLC10A2*, and *ZCWPW1*) and approximately 100 other provisional and confirmed genes and regions that were identified by candidate gene and GWAS approaches in studies of AD and AD-related traits (Saunders et al., 1993; Farrer et al., 2000; Meng et al., 2006; Rogaeva et al., 2007; Vardarajan et al., 2012; Lambert et al., 2013; Reitz et al., 2013b; Jun et al., 2014; Logue et al., 2014; Wetzel-Smith et al., 2014; Jun et al., 2016; Chung et al., 2017; Mez et al., 2017) (**Supplementary Table S1**). Nimblegen probes (Roche Nimblegen, Madison, WI, United States) were generated to cover all non-repetitive exonic, intronic, and intergenic sequence and 5,000 bp upstream and 1,000 bp downstream of gene boundaries including all isoforms totaling approximately nine Mb of genomic sequence. Only exons were targeted for *SLC10A2* and *COBL* because these associations (Mez et al., 2017) were not known at the time the capture design was proposed and the limited amount of genomic sequence that could be added to the capture at this stage. The capture design included 10,906 capture targets and had 92.7% estimated coverage of 122 targeted regions, with gaps due to repetitive sequence.

Samples were assessed for volume and concentration by the Genome Center using either a Qubit or a VarioSkan assay prior to sequencing. All but eight had >250 ng starting material. The sequencing was done in two waves. The first wave included 667 samples from the MIRAGE and GenerAAtions cohorts. Libraries were captured in sets of 66 and 67 samples per pool and each pool was run in two lanes of an Illumina Hiseq2500 1T platform. The remaining discovery cohort samples were sequenced in the second wave using the same capture probes in pools of 90 samples each, and each sample was run on 2 lanes of an Illumina HiSeq4000 platform. Valid sequence data were available for a discovery cohort including 489 cases and 472 controls. The median number of reads per sample was 14,322,643 (range 6,175,120–25,567,585). The median number of reads was greater for the samples run on the HiSeq4000 platform (median reads/sample for batch 1 = 12,809,719, median reads/sample for batch 2 = 16,804,253). In batch 1, the number of reads/sample for the MIRAGE Study samples (median = 12,331,728) was significantly less than for the GenerAAtions samples (median = 17,217,602, *P* = 3.51 × 10^-5^). The number of reads per sample for the second batch of sequencing did not vary by cohort (*p* = 0.17). Importantly, the number of reads per sample was not associated with AD status in either batch or in the combined discovery sample (all *p* > 0.3). Across samples, the median percentage of bases with more than 10 reads was 94.60 and the mean coverage depth was 155.7.

### Sanger Sequencing

Genotyping for the *ABCA7* deletion polymorphism rs567222111 was performed in the replication sample by GENEWIZ (GENEWIZ LLC, South Plainfield NJ, United States^2^) using bi-directional Sanger sequencing. Sequencing was repeated for samples that did not yield a reliable genotype call in the first run. Validity of the Sanger sequencing assay was demonstrated by verifying genotype calls for 10 samples which had been identified as having the deletion by targeted-sequencing.

### Data Processing and Quality Control

The 126 bp paired-end reads were aligned to the GRCh37 +Decoy reference with BWA MEM version 0.7.10-r789. Variant genotypes were jointly called within the targeted regions using the GATK 3.7 pipeline. The “best practices” pipeline included steps for duplicate removal, local realignment near indels, base quality score recalibration, and variant quality score recalibration. GATK yielded calls for 230,595 variants. Annotation of the variants was performed with SnpEff and SnpSift version 4.3i (Cingolani et al., 2012). According to SnpEff, these variants mapped to 151 protein-coding genes. Variants that were not assigned a “PASS” rating by GATK (*n* = 11,808) were excluded from association analyses. We also excluded variants in the HLA region (*n* = 24,297) due to difficulties in mapping the repetitive sequence and variants in the *APOE* region (*n* = 197) due to difficulties discerning associations in this region that are independent of *APOE* (Jun et al., 2017). However, we did use sequence calls to derive *APOE* isoform genotypes for QC purposes (see description below). Another 5,147 variants occurring only in subjects with missing phenotype information were excluded. After these filtering steps, 189,145 variants remained. From this point forward, the pipeline differed for single variant association tests and the gene based tests. For the single variant test, variants observed only once (*n* = 66,278) were excluded. Genotypes with quality scores <30 were set to missing and variants with a missing rate of >20% were excluded (*n* = 18,526). After these filtering steps, there remained 104,341 variants for analyses. For the gene based tests, we included singleton variants but excluded variants with a mapping quality of less than 30 (*n* = 3,748 of 189,145). We excluded variants with minor allele frequency (MAF) in the discovery cohort >5% (32,935). One hundred seventy-three of these variants labeled as “High Impact” according to SNPeff (includes LOF variants and deletions) and 1,079 missense SNPs predicted to be possibly or probably damaging according to Polyphen2 (Adzhubei et al., 2010) were included in the gene-based analyses.

As a quality control check, we compared genotypes for *APOE* and two rare *AKAP9* missense variants (rs144662445 and rs149979685) in MIRAGE cohort subjects that were generated previously by direct genotyping to those derived from targeted sequencing. The two methods agreed for 236 of 237 *APOE* genotype calls. Among 190 subjects with overlapping genotype and sequencing data for the *AKAP9* variants, rare variant calls in three individuals (each with both variants) were concordant.

### Statistical Analysis

We applied a hypothesis-driven four-stage design which prioritized variants most likely to have high impact on transcript structure or function in order to minimize the penalty associated with performing more than 100,000 tests. Specifically, variants were selected for analysis if they were (1) predicted to result in loss of function according to the SNPeff annotation, which includes nonsense (stop site) and splice site variants, out of frame deletions/insertions, and large exon-removing deletions (MacArthur et al., 2012), (2) predicted to be a missense variant according to SNPeff, and (3) within 50 base pairs (bp) of transcription start sites (position determined via the Eukaryotic Promotor Database^3^). We then examined (4) all variants (intronic and exonic) regardless of potential impact. To avoid model instability that can occur with logistic or GEE or mixed models when applied to rare variants, association of AD with individual variants was evaluated using a X^2^ case:control allele test without continuity correction as implemented in PLINK v1.9 (Chang et al., 2015). For particular variants of interest identified in the allele test, we additionally checked for bias due to relatedness within the MIRAGE cohort as well as potential effects due to population substructure by computing a WALD test using a logistic mixed model in the R GMMAT package (Chen et al., 2016) including as covariates the first three principle components (PCs) for ancestry. The GMMAT package incorporates information from the relationship matrix which we computed from the genetic data in PLINK v1.9 based on 4,569 common (MAF>5%) variants from the sequence data remaining after trimming for LD (plink filter –indep-pairwise 5 20.04). PCs were also computed based on common LD-trimmed SNPs using PLINK. Gene based tests were performed for the 151 protein coding genes (as identified by the SNPeff annotation) using the variable threshold burden test (Price et al., 2010) and the collapsing burden test methods (Li and Leal, 2008) implemented in EPACTS^4^ which incorporates information about related subjects in the sample. The correlation matrix for related subjects for the gene based test was estimated from the sequence data. LD estimates for 1000 genomes data were obtained using LDlink^5^. LD estimates for the sequencing results from the AD cohort were estimated using PLINK v1.9 with the –rsq dprime option.

This study, involving use of repository data and biospecimens, was approved by the Boston University Institutional Review Board.

## Results

### Genes Previously Associated With AD in AAs

#### LOF Variants

In the seven genes previously associated with AD in AAs, nominally significant associations were observed for a rare LOF variant in *MS4A6A* observed only in controls (rs140130948, *p* = 0.013) and an 11 bp *ABCA7* deletion (rs567222111, *OR* = 3.57, *p* = 0.038) which had an estimated allele frequency (AF) of 1.1% in cases and 0.32% in controls (**Table 2**). This association remained significant in the mixed model adjusting for relatedness within the sample and including PCs for ancestry (*OR* = 3.65, *p* = 0.049). This deletion had a stronger impact on AD risk than the more common 44 bp *ABCA7* deletion (rs142076058) which was previously reported to be associated with AD in an AA cohort (*OR* = 1.81) (Cukier et al., 2016) but not in our sample (*OR* = 1.27, *p* = 0.16). The evidence for association with rs567222111 in the replication sample was not significant, but had the same effect direction (*OR* for the deletion = 1.84, *p* = 0.22), and the significance in the combined discovery and replication samples was greater than in the discovery sample alone (*OR* = 2.42, *p* = 0.022). No LOF variants were observed in *AKAP9*, *COBL*, *PTK2B*, *SLC10A2, or ZCWPW1*.

**Table 2:** Association between AD and loss of function (LOF) variants observed in African American AD genes.

Gene	Ch.	BP	rsID	Effect Allele	% AFR	% Cases	% Ctrls	Alt. Allele	*OR*	*P*
*MS4A6A*	11	59,939,727	rs140130948	G	0.15	0.00	0.64	C	0.00	0.013
*ABCA7*	19	1,044,707	rs567222111	G	0.83	1.13	0.32	GGGGCACCTGGT	3.57	0.038
*ABCA7*	19	1,041,352	rs3752229	G	0.15	0.41	0.95	A	0.43	0.15
*ABCA7*	19	1,056,244	rs113809142	G	0.00	0.00	0.21	T	0.00	0.15
*ABCA7*	19	1,046,906	rs142076058	G	6.73	9.20	7.42	GCTGCGGGACAC CATGCGCGCCAT GGGGCTCAGCC GCGCGGTGCT	1.27	0.16
*ABCA7*	19	1,058,727	rs556286113	T	0.15	0.20	0.00	C	NA	0.16
*ABCA7*	19	1,043,395	rs77403558	T	0.30	0.31	0.11	A	2.90	0.33
*MS4A6A*	11	59,946,302	rs598862	C	30.18	25.87	26.69	T	0.96	0.68
*MS4A6A*	11	59,940,500	rs138650483	T	0.00	0.10	0.11	C	0.97	0.98


Effect allele represents the minor allele; % AFR represents the estimated effect allele frequency in the 1000 Genomes African cohort; % Cases represents the estimated effect allele frequency in AD cases; % Controls represents the estimated effect allele frequency in controls.

#### Missense Variants

Association tests were nominally significant for 14 of 172 missense variants tested in the seven genes previously associated with AD in AAs including a common SNP in *ABCA7* (rs5985184, *p =* 0.0043) and the rare missense variants in *AKAP9*, rs149979685 (*OR* = 10.73, *p* = 0.0046) and rs144662445 (*OR* = 6.35, *p* = 0.0054), previously identified in a sample that overlaps substantially with the discovery cohort in this study (Logue et al., 2014) (**Table 3**). Our analysis also confirmed the previously reported association for one of the common *ABCA7* missense SNPs noted in N’Songo et al., 2017 (rs3764647, *OR* = 1.29 for minor allele, p = 0.017), but not the rare coding variant (rs3752239, *OR* = 0.39, *p* = 0.24). Consistent with prior results (Logue et al., 2014), the association with the rare *AKAP9* variants was significant in a mixed model which adjusted for relatedness within the sample with ancestry PCs as covariates (for rs149979685 *OR* = 10.53, *p* = 0.025 and for rs144662445 *OR* = 6.25, *p* = 0.016).

**Table 3:** Nominally significant missense variants in the 7 AA-AD genes.

Gene	Ch.	BP	rsID	Effect Allele	% AFR	% Cases	% Ctrls	Alt. Allele	*OR*	*P*
*ABCA7*	19	1,047,336	rs59851484	A	11.88	14.83	10.49	G	1.49	0.0043
	19	1,058,635	rs73505232	T	14.30	16.05	12.18	C	1.38	0.015
	19	1,044,712	rs3764647	G	25.72	26.24	21.60	A	1.29	0.017
	19	1,056,492	rs3752246	G	1.06	3.48	5.72	C	0.59	0.019
	19	1,043,748	rs3752232	G	27.08	27.20	23.20	A	1.24	0.044
	19	1,057,335	rs538930513	A	0.30	0.41	0.00	G	NA	0.049
*AKAP9*	7	91,732,110	rs149979685	T	0.45	1.13	0.11	C	10.73	0.0046
	7	91,709,085	rs144662445	G	0.53	1.33	0.21	A	6.35	0.0054
	7	91,726,202	rs78351282	A	2.80	3.27	1.59	G	2.10	0.017
	7	91,726,604	rs34956633	G	4.92	5.11	7.54	A	0.66	0.029
	7	91,712,808	rs149946443	A	1.13	0.51	1.48	G	0.34	0.032
	7	91,630,603	rs143894795	C	0.76	0.31	1.06	G	0.29	0.044
*SLC10A2*	13	103,718,308	rs55971546	T	0.30	0.31	1.06	C	0.29	0.044


Effect allele represents the minor allele; % AFR represents the estimated effect allele frequency in the 1000 Genomes African cohort; % Cases represents the estimated effect allele frequency in AD cases; % Controls represents the estimated effect allele frequency in controls.

#### Regulatory Variants

We also examined potentially regulatory variants in the AD genes implicated in AAs. Association was tested with variants in regulatory regions for the two primary *AKAP9* isoforms. One variant identified near the TSS of the shorter isoform was not associated with AD (*p* = 0.66). Significant association was identified with a rare nine bp deletion (rs371245265) located near the TSS for the longer *AKAP9* isoform (*OR* for the deletion = 6.37, *p* = 0.0053). Prompted by the similarity of allele frequencies between this deletion and the previously identified coding AD risk variants (rs144662445 and rs149979685), we checked the 1000 genomes phase 3 African population data and confirmed high LD between rs371245265 and both rs144662445 (*r*^2^ = 0.86) and rs149979685 (*r*^2^ = 1). Consistent with this information, all 17 discovery sample subjects with the rs371245265 deletion were also carriers of the rs144662445 minor allele, and 14 of these subjects were also carriers of the rs149979685 minor allele. As noted for rs149979685, the association with rs371245265 remained significant in a model adjusting for relatedness and ancestry (*OR* = 6.30, *p* = 0.016). Nominally significant associations were also observed with three common potentially regulatory SNPs in *ZCWPW1*. The most significant of these three was rs10693652, a 2 bp deletion which was more common in controls than cases (*OR* for the deletion = 0.75, *p* = 0.0042). The sole *ABCA7* variant and 13 *PTK2B* variants located in TSSs were not associated with AD. Regulatory variants in *COBL* and *SLC10A2* could not be evaluated because the custom capture design for these loci included exons only.

#### Other Variants

Examination of the full complement of variation in these genes (*n* = 4,325) including 342 variants in *ABCA7*, 1,445 in *AKAP9*, 167 in *COBL*, 204 in *MS4A6A*, 1,874 in *PTK2B*, 37 variants in *SLC10A2*, and 256 variants in *ZCWPW1* revealed many nominally significant associations (**Table 4**). The most significant association was observed with a rare SNP in *PTK2B* (rs115828696, MAF = 0.0020 in AD cases and 0.18 in controls) which was protective (*OR* for the minor allele *A* = 0.11, *p* = 0.00041). A strong protective effect was also identified with a common SNP in *ZCWPW1* (*OR* = 0.67, *p* = 0.0013). Genotypes were not available for several of the previously implicated AA-specific risk SNPs including *ABCA7* rs115550680 (Reitz et al., 2013a,b) which is located in a repetitive region and was not captured by the design. The *COBL* rs112404845 and *SLC10A2* rs16961023 variants (Mez et al., 2017) are outside of the coding regions and, thus, were not assessed.

**Table 4:** Top-ranked association results in previously established AA AD-risk genes.

Gene	Ch.	BP	rsID	Eff. All.	% AFR	% Cases	% Ctrls	Alternate Allele	*OR*	*P*
*ABCA7*	19	1,050,007	.	–	NA	4.63	2.16	C	2.20	0.0042
	19	1,047,336	rs59851484	A	11.88	14.83	10.49	G	1.49	0.0043
	19	1,049,991	.	–	NA	5.03	2.54	CCTCCCTGT GAGCCCCCC ACCACTT	2.03	0.0065
	19	1,043,260	rs58262414	G	11.72	14.62	10.59	T	1.45	0.0079
	19	1,042,598	rs147599642	A	11.95	14.62	10.70	AAT	1.43	0.0098
*AKAP9*	7	91,570,040	rs557208555	C	0.53	1.13	0.11	A	10.73	0.0046
	7	91,591,230	rs114789310	A	0.53	1.13	0.11	G	10.73	0.0046
	7	91,663,031	rs183984025	T	0.53	1.13	0.11	C	10.73	0.0046
	7	91,732,110	rs149979685	T	0.45	1.13	0.11	C	10.73	0.0046
	7	91,590,199	rs564709734	G	0.53	1.13	0.11	A	10.70	0.0046
*COBL*	7	51,085,149	rs150183973	A	1.81	1.94	0.74	T	2.65	0.023
	7	51,098,567	rs142060269	G	NA	44.07	39.07	GTCT	1.23	0.026
	7	51,098,849	rs62448278	A	53.03	48.98	44.17	G	1.21	0.035
	7	51,138,814	rs1295400	T	6.28	7.67	5.30	C	1.49	0.035
*MS4A6A*	11	59,939,727	rs140130948	G	0.15	0.00	0.64	C	0.00	0.013
	11	59,945,018	rs146080691	A	0.15	0.00	0.32	G	0.00	0.077
	11	59,943,683	rs183204829	T	0.00	0.00	0.32	C	0.00	0.078
	11	59,950,406	rs577683097	A	0.15	0.00	0.32	G	0.00	0.078
	11	59,940,141	rs186332028	C	0.68	0.20	0.74	T	0.27	0.085
*PTK2B*	8	27,253,935	rs115828696	G	0.68	0.20	1.80	A	0.11	0.00041
	8	27,268,750	rs3757908	T	1.06	3.89	1.91	C	2.08	0.0099
	8	27,276,111	rs891392	C	1.06	3.89	1.91	T	2.08	0.0099
	8	27,272,298	rs144318332	G	4.31	2.05	4.03	C	0.50	0.011
	8	27,280,472	rs77318377	A	4.31	2.05	4.03	G	0.50	0.011
*SLC10A2*	13	103,718,308	rs55971546	T	0.30	0.31	1.06	C	0.29	0.044
	13	103,718,824	rs16961281	A	13.16	10.84	8.16	G	1.37	0.045
	13	103,719,056	rs7987433	C	23.22	23.21	19.81	T	1.22	0.070
	13	103,697,359	rs199983061	T	0.15	0.20	0.64	C	0.32	0.14
	13	103,697,329	rs41281676	A	4.23	5.11	3.81	G	1.36	0.17
*ZCWPW1*	7	100,002,772	rs76913697	G	11.57	13.19	18.54	A	0.67	0.0013
	7	100,026,415	rs10693652	TCA	29.43	25.56	31.46	T	0.75	0.0042
	7	100,028,484	rs6962151	C	29.43	25.56	31.46	T	0.75	0.0042
	7	100,025,564	rs67196635	C	29.43	25.61	31.45	T	0.75	0.0047
	7	100,014,313	rs6957928	A	12.93	15.24	19.85	G	0.73	0.0078


Effect allele represents the minor allele; % AFR represents the estimated effect allele frequency in the 1000 Genomes African cohort; % Cases represents the estimated effect allele frequency in AD cases; % Controls represents the estimated effect allele frequency in controls; “–“ indicates a deletion; “.” indicates a variant without an annotated rsID; NA indicates the variant is not present in 1000 Genomes.

### Genes Previously Associated With AD in Other Ancestry Groups

Of the 104,341 variants observed in all targeted regions that were tested for association with AD, 29 were annotated as LOF variants. Only the previously noted *MS4A6A* and *ABCA7* variants (rs140130948 and rs567222111) were nominally significant (**Table 5**). The most significant association findings among 1,067 missense variants were obtained with five common highly correlated variants in *PILRB* that showed a protective effect (0.0010 < *p* < 0.0017; estimated OR for minor alleles varied from 0.65 to 0.67; **Table 6**). Restricting the analysis to potentially regulatory variants, a protective common indel near the TSS of *ZCWPW1* (rs10693652, *OR* for the minor allele = 0.75, *p* = 0.0042) and the rare risk indel near the TSS of *AKAP9* (rs536714523) noted above were the most significant of the 223 variants tested (**Table 7**). Finally, examination of the entire set of 104,341 variants identified in the targeted sequencing experiments yielded significant associations with multiple loci (**Table 8**), most notably a rare protective variant in *F5* (rs2027885, OR for minor allele *A* = 0.053, *p* = 6.40 × 10^-5^), a gene that was previously associated with a MRI measure of hippocampal atrophy (Melville et al., 2012), and two common variants in *KIAA0196* (*p* < 8.6 × 10^-5^; **Table 8**). Out of the 151 protein-coding genes, nominally significant gene-based associations were found with six genes using the CMC test and with three genes using the VT test (**Table 9**). The most significant of these results was *KANSL1* (*p* = 0.013). None of the seven previously established AD risk genes in AAs were significant (*p* > 0.05).

**Table 5:** Top loss of function variants from all sequenced genes (out of 29 LOF variants examined).

Gene	Ch.	BP	rsID	Effect Allele	% AFR	% Cases	% Ctrls	Alt. Allele	*OR*	*P*
*MS4A6A*	11	59,939,727	rs140130948	G	0.15	0.00	0.64	C	0.00	0.013
*ABCA7*	19	1,044,707	rs567222111	G	0.83	1.13	0.32	GGGGCACCTGGT	3.57	0.038
*CD33*	19	51,738,933	rs273621	C	2.27	0.72	1.59	T	0.45	0.072
*ACE*	17	61,563,661	rs4330	C	41.30	40.11	43.61	A	0.87	0.124
*ABCA7*	19	1,041,352	rs3752229	G	0.15	0.41	0.95	A	0.43	0.146


Effect allele represents the minor allele; % AFR represents the estimated effect allele frequency in the 1000 Genomes African cohort; % Cases represents the estimated effect allele frequency in AD cases; % Controls represents the estimated effect allele frequency in controls.

**Table 6:** Top missense variants from all sequenced genes (out of 1,067 missense variants examined).

Gene	Ch.	BP	rsID	Effect Allele	% AFR	% Cases	% Ctrls	Alt. Allele	*OR*	*P*
*PILRB*	7	99,956,444	rs11761306	G	NA	11.86	17.13	A	0.65	0.0010
*PILRB*	7	99,956,436	rs11771799	C	7.03	11.89	17.09	T	0.65	0.0012
*PILRB*	7	99,956,439	rs35986051	C	7.03	11.89	17.09	T	0.65	0.0012
*PILRB*	7	99,955,866	rs61735533	A	10.14	12.07	17.27	G	0.66	0.0013
*ABCA7*	19	1,047,336	rs59851484	A	11.88	14.83	10.49	G	1.49	0.0043
*AKAP9*	7	91,732,110	rs149979685	T	0.45	1.13	0.11	C	10.73	0.0046
*AKAP9*	7	91,709,085	rs144662445	G	0.53	1.33	0.21	A	6.35	0.0054
*KIAA0196*	8	126,091,036	rs143719918	T	0.23	1.02	0.11	C	9.74	0.0077
*ECHDC3*	10	11,797,500	rs35986488	A	3.86	3.78	6.36	G	0.58	0.0100


Effect allele represents the minor allele; % AFR represents the estimated effect allele frequency in the 1000 Genomes African cohort; % Cases represents the estimated effect allele frequency in AD cases; % Controls represents the estimated effect allele frequency in controls; NA indicates the variant is not present in 1000 Genomes.

**Table 7:** Top potentially regulatory variants from all sequenced genes (out of 223).

Gene	Ch.	BP	rsID	Effect Allele	% AFR	% Cases	% Ctrls	Alt. Allele	*OR*	*P*
*ZCWPW1/MEPCE*	7	100,026,415	rs10693652	TCA	29.43	25.56	31.46	T	0.75	0.0042
*AKAP9*	7	91,570,197	rs536714523	T	0.45	1.34	0.21	TGGCGGCGGC	6.37	0.0053
*ZCWPW1*	7	100,014,846	rs73161762	T	1.13	3.17	5.30	C	0.59	0.020
*ZCWPW1/MEPCE*	7	100,027,339	rs74460138	G	14.29	16.36	20.44	C	0.76	0.021
*SNX6*	14	35,099,305	rs562903264	A	0.00	0.51	0.00	G	NA	0.028
*KANSL1*	17	44,302,765	rs187276691	A	0.15	0.72	0.11	G	6.80	0.038
*NSMCE2/KIAA0196*	8	126,104,130	rs76575464	A	19.74	18.51	14.97	C	1.29	0.038
*CELF1*	11	47,574,654	rs575641108	CGCCGCT	0.15	0.50	0.00	C	NA	0.047
*BZRAP1/BZRAP1-AS1/MIR142*	17	56,406,133	rs374170329	G	0.23	0.41	0.00	C	NA	0.049


Effect allele represents the minor allele; % AFR represents the estimated effect allele frequency in the 1000 Genomes African cohort; % Cases represents the estimated effect allele frequency in AD cases; % Controls represents the estimated effect allele frequency in controls.

**Table 8:** Top variants from all genes (out of 104,341 variants examined).

Gene	Ch.	BP	rsID	Eff. All.	% AFR	% Cases	% Ctrls	Alt. Allele	*OR*	*P*
*F5*	1	169,535,038	rs2027885	A	0.61	0.10	1.91	G	0.053	6.40E-05
*KIAA0196*	8	126,097,380	rs79300936	A	29.73	30.06	22.14	G	1.51	7.83E-05
*KIAA0196*	8	126,097,473	rs7832481	G	29.80	29.96	22.08	A	1.51	8.50E-05
*KIAA0196*	8	126,066,723	rs7817741	A	2.19	6.44	11.12	C	0.55	0.00028
*KIAA0196*	8	126,073,786	rs2272729	A	2.12	6.44	11.12	G	0.55	0.00028
*KIAA0196*	8	126,093,882	rs7817303	A	18.31	20.25	14.09	G	1.55	0.00035
*PTK2B*	8	27,253,935	rs115828696	G	0.68	0.20	1.80	A	0.11	0.00041
*PLXNC1*	12	94,696,160	rs189295092	–	2.12	5.46	2.29	C	2.46	0.00043
*SORL1*	11	121,330,087	rs3862606	G	19.82	18.51	25.11	A	0.68	0.00046
*PILRB*	7	99,965,328	rs11284139	G	10.14	11.96	17.58	GA	0.64	0.00051


Effect allele represents the minor allele; % AFR represents the estimated effect allele frequency in the 1000 Genomes African cohort; % Cases represents the estimated effect allele frequency in AD cases; % Controls represents the estimated effect allele frequency in controls; “–“ indicates a single bp deletion.

**Table 9:** Nominally significant gene-based burden tests of association with AD.

Test	Gene		Start (bp)	End (bp)	Num. Variants Included	*P*
CMC	*KANSL1*	17	44,112,733	44,249,388	124	0.013
	*TRIM35*	8	27,168,348	27,168,671	12	0.018
	*PLEKHM1*	17	43,515,240	43,559,893	115	0.029
	*MS4A6E*	11	60,102,408	60,105,226	9	0.038
	*PTK2B*	8	27,168,348	27,315,954	163	0.047
	*PILRA*	7	99,971,735	100,001,863	80	0.053
VT	*CNN2*	19	1,036,201	1,043,455	139	0.017
	*PLEKHM1*	17	43,515,240	43,559,893	115	0.045
	*TRIM35*	8	27,168,348	27,168,671	12	0.049


## Discussion

We performed targeted gene sequencing in an AA cohort containing 489 AA AD cases and 472 cognitively normal controls and found evidence of association with several novel variants in genes that were previously implicated with AD risk in AAs including a deletion causing LOF of *ABCA7* (rs567222111). We subsequently genotyped this deletion in an independent cohort containing 484 AD cases and 484 controls, and the association with this large effect variant (*OR* = 2.42) became more significant in the combined sample. Another notable novel association was identified with a rare 9 bp. deletion (rs371245265) located near the TSS of *AKAP9*. We also confirmed previously reported associations with missense variants in *ABCA7* (rs3764647) and *AKAP9* (rs149979685 and rs144662445). Gene-based tests of aggregated rare variants yielded several associations, most significantly with *KANSL1*, *CNN2*, and *TRIM35*.

The association with the *AKAP9* regulatory region variant rs371245265 calls into question whether the previously identified *AKAP9* missense variants (rs149979685 and rs144662445) are causally related to AD because all of these variants are in high LD. Previous analysis of the background haplotype harboring rs149979685 and rs144662445 and spanning an 800 kb region including five genes showed that no other coding variants could explain the association with these *AKAP9* missense variants (Logue et al., 2014). However, it remains possible that the rs371245265 variant has a regulatory effect on *AKAP9* expression, and this variant alone or in conjunction with the missense variants, could underlie the observed association with AD risk. Because these three rare variants most often co-occur, it is unlikely that the potentially causal effects of these variants will be disentangled by epidemiological studies. Recently, we observed significantly higher phosphorylation and greater post-translational modifications of Tau protein in lymphoblastoid cells from subjects having at least one of the missense variants, a finding that was independent of the disease status of the cell donors (Ikezu et al., 2018). However, since these subjects also have the potentially regulatory variant, experimental studies will be necessary to determine whether this variant does indeed have a regulatory effect and in particular which of the three variants account for the observed effect on Tau phosphorylation.

Our observed novel association with a rare 11 bp loss of function frameshift deletion (rs567222111, Leu396fs) in a gene encoding one of the ATP-binding cassette transporter proteins (*ABCA7*) adds to a growing list of AD-associated LOF mutations in this gene (Farrer, 2015). The most remarkable of these is a 7 bp deletion, causing a frameshift mutation (Glu709fs) that was detected in 11 out of 772 unrelated patients but not in 757 controls from the Flanders region of northern Belgium (Cuyvers et al., 2015). Association of this mutation with AD has also been observed in several other European ancestry populations (Steinberg et al., 2015). Cukier et al reported association of AD and a relatively common 44 bp LOF deletion in *ABCA7* (rs142076058, Ser587fs, *OR* = 2.13) in an AA cohort that is non-overlapping with our study sample (Cukier et al., 2016). This deletion was observed in the current study, but had a smaller effect on AD risk (*OR* = 1.27, *p* = 0.16). Of note, the frameshift mutation identified in our study occurs earlier in the amino acid sequence (position 396) than the Belgian (position 709) or other AA (position 587) frameshift mutations and thus may yield a more seriously impaired protein than these other mutations, but this will have to be confirmed experimentally.

Surprisingly, expanding the analyses from the relatively small set of genes that were implicated in previous studies of AAs to the larger set of AD genes that were established in other populations yielded relatively few significant results, the most significant of which is a rare protective variant (rs2027885) in the gene encoding the blood clotting protein Factor 5 *(F5*, *OR* = 0.053, *p* = 6.40 × 10^-5^). A GWAS of a brain MRI measure of hippocampal atrophy in a MIRAGE Study sample composed primarily of AD and control subjects of European ancestry and a smaller group of AAs (many of which are included in this study) found genome-wide significant association with several common SNPs spanning portions of *F5* and its immediate neighbor, *SELP*, that was supported by evidence in both populations (Melville et al., 2012). Although there is scant genetic evidence linking *F5* to AD, it has been shown that factor V activating protein in Russell’s viper venom destabilizes amyloid-β aggregates as revealed from a thioflavin T assay (Bhattacharjee and Bhattacharyya, 2013).

Our findings contrast those of another recent exome sequencing study of AD in a smaller sample of AAs (198 AD cases and 304 controls) which focused exclusively on 20 loci reaching genome-wide significance in a very large GWAS of European ancestry cohorts (N’Songo et al., 2017). The previous study found nominally significant associations with two variants in *ABCA7* (rs3764647 and rs3752239) and in gene-based tests of coding variants in *MS4A6A*, *PTK2B*, and *ZCWPW1*. We observed association with rs3764647 (*p* = 0.017), but did not replicate the association with rs3752239 or the gene-based associations. On the other hand, gene-based tests of aggregated rare variants in *KANSL1*, *TRIM35*, *MS4A6E*, and *PILRA* were nominally significant in our study. Differences in findings may be due in part to the use of exome sequencing by N’Songo et al. (2017) versus sequencing of complete gene regions in our study which allowed detection of association with potentially functional variants in regulatory regions and introns that influence transcription and splicing, as well as with structural variants that span non-coding regions.

Our findings should be interpreted cautiously. None of our findings remain significant after correcting for the total number of tests performed in the study. Our sample size was not large enough to detect associations with rare variants exerting modest effects with experiment-wide significance. Also, our primary analyses of individual variants did not account for the correlated structure of our dataset which included many related individuals. Our study highlights the difficulty of obtaining statistically significant results with rare variants, especially those with frequencies less than 1%. It is essential to replicate our findings in independent AA samples, and sufficiently large samples will become available eventually through the efforts of large consortia including the Alzheimer’s Disease Genetics Consortium and Alzheimer’s Disease Sequencing Project. In addition, experimental studies are needed to establish functionally relevant roles of these genes and variants in AD pathogenesis.

With these concerns in mind, the goal of this study was to identify variants with supporting genetic evidence and predicted functional impact for examination in relevant biological systems. Given the previously identified relationship between loss of function mutations in *ABCA7* and AD (Cuyvers et al., 2015; Farrer, 2015; Steinberg et al., 2015; Cukier et al., 2016) and genetic and biological evidence for a role of rare *AKAP9* variants in AD (Logue et al., 2014; Ikezu et al., 2018), the novel *ABCA7* coding. region deletion (rs567222111) and the potentially regulatory *AKAP9* deletion (rs371245265) are the most compelling findings for future studies.

## Data Availability

The unprocessed sequence data generated for this study can be found in the National Institute on Aging Genetics of Alzheimer’s Disease Data Storage Site (https://www.niagads.org/).

## Author Contributions

MWL, DL, LAF, and KLL contributed to the study design. JF cleaned and processed the sequence data. IS extracted and performed quality control on the DNA specimens used for sequencing and genotyping. MWL and DL performed analyses of the data and prepared the results for presentation. MWL and LAF drafted the manuscript. All authors contributed to the editing and revision of the manuscript.

## Conflict of Interest Statement

The authors declare that the research was conducted in the absence of any commercial or financial relationships that could be construed as a potential conflict of interest.

## Footnotes

1 http://genome.wustl.edu

2 http://www.genewiz.com

3 http://epd.vital-it.ch/index.php

4 http://genome.sph.umich.edu/wiki/EPACTS

5 https://analysistools.nci.nih.gov/LDlink/

## Supplementary Material

TABLE S1 Targeted regions including provisional and confirmed loci associated with AD and related phenotypes.
